# Inhibition of calcium-calmodulin-dependent phosphodiesterase (PDE1) suppresses inflammatory responses

**DOI:** 10.1016/j.mcn.2019.103449

**Published:** 2019-11-23

**Authors:** Jennifer J. O'Brien, James P. O'Callaghan, Diane B. Miller, Suman Chalgeri, Lawrence P. Wennogle, Robert E. Davis, Gretchen L. Snyder, Joseph P. Hendrick

**Affiliations:** a Intra-Cellular Therapies, Inc., The Alexandria Center for Life Sciences, 430 East 29th St Suite 900, New York, NY 10016, United States of America; b Centers for Disease Control and Prevention, National Institute for Occupational Safety and Health, Morgantown, WV 26505, United States of America

**Keywords:** Cyclic nucleotide, Calcium, Microglia, Migration, VASP, Phosphorylation

## Abstract

A novel, potent, and highly specific inhibitor of calcium-calmodulin-dependent phosphodiesterases (PDE) of the PDE1 family, ITI-214, was used to investigate the role of PDE1 in inflammatory responses. ITI-214 dose-dependently suppressed lipopolysaccharide (LPS)-induced gene expression of pro-inflammatory cytokines in an immortalized murine microglial cell line, BV2 cells. RNA profiling (RNA-Seq) was used to analyze the impact of ITI-214 on the BV2 cell transcriptome in the absence and the presence of LPS. ITI-214 was found to regulate classes of genes that are involved in inflammation and cell migration responses to LPS exposure. The gene expression changes seen with ITI-214 treatment were distinct from those elicited by inhibitors of other PDEs with anti-inflammatory activity (e.g., a PDE4 inhibitor), indicating a distinct mechanism of action for PDE1. Functionally, ITI-214 inhibited ADP-induced migration of BV2 cells through a P2Y12-receptor-dependent pathway, possibly due to increases in the extent of cAMP and VASP phosphorylation downstream of receptor activation. Importantly, this effect was recapitulated in P2 rat microglial cells in vitro, indicating that these pathways are active in native microglial cells. These studies are the first to demonstrate that inhibition of PDE1 exerts anti-inflammatory effects through effects on microglia signaling pathways. The ability of PDE1 inhibitors to prevent or dampen excessive inflammatory responses of BV2 cells and microglia provides a basis for exploring their therapeutic utility in the treatment of neurodegenerative diseases associated with increased inflammation and microglia proliferation such as Parkinson's disease and Alzheimer's disease.

## 1. Introduction

Neuroinflammation has been suggested as a contributory feature in many neurological and neurodegenerative diseases. Activation of microglia, brain resident immune cells, appears to be an important participating event in inflammatory processes in diseases of the central nervous system. Gaining a greater appreciation for mechanisms underlying microglia activation may yield new therapeutic options for treating these diseases. Inhibition of cyclic nucleotide phosphodiesterases is regarded as one promising therapeutic approach for controlling microglia mediated neuroinflammation. Others have shown that increasing microglial cAMP levels by means of isoproterenol, forskolin, or non-hydrolysable 8-Br-cAMP attenuates LPS-induced production of tumor necrosis factor (TNF) and interleukin-6 (IL6) (Consonni et al., 2011). The PDE1 phosphodiesterases (i.e., PDE1A, 1B, and 1C isoforms) are dual cAMP/cGMP phosphodiesterase activated by calcium/calmodulin, and thus, function to dampen cyclic nucleotide signaling in excitatory cells such as neurons, cardiac myocytes, and possibly microglia. As human and mouse microglial cells express PDE1B, one of the 3 main isoforms of this enzyme, at significant levels (Zhang et al., 2014), we reasoned that inhibition of PDE1 in inflammatory cells, particularly microglia, might alter cyclic nucleotide signaling and be therapeutic in diseases associated with excessive inflammatory signaling. To this end, we used a microglia-like cell line, immortalized murine microglial cell line BV2, to model certain microglial cellular functions in vitro that are mediated by altered cyclic nucleotide signaling and responses to PDE1 inhibition. Selected findings were replicated in murine primary microglia.

We have developed a specific PDE1 inhibitor, ITI-214, that has submicromolar affinity for all three PDE1 isoforms. ITI-214 is cell permeant and enters the brain, where PDE1A, 1B, and 1C enzymes are present, and exerts pro-cognitive effects in animal memory paradigms (Li et al., 2016; Snyder et al., 2016). More recently, ITI-214 has been shown to acutely enhance cardiac function in PDE1C-expressing mammals (Hashimoto et al., 2018). Here, we describe the anti-inflammatory activity of ITI-214 in vitro in BV2 cells activated by LPS. We have measured the induction of cytokine and chemokine expression by LPS using an enzyme-linked immunosorbent assay (ELISA) and by quantitative analysis of corresponding mRNAs (qPCR). Using RNA profiling (RNA-Seq), we have analyzed the impact of ITI-214 on the BV2 transcriptome in the absence and the presence of LPS. Gene ontology analysis of the results revealed effects of ITI-214 on the BV2 transcriptome distinct from a PDE4-inhibitor comparator and predicted a role for PDE1 in modulating cell migration and chemotaxis. We show that inhibition of PDE1 by ITI-214 dose dependently interrupts ADP induced chemotaxis through a biochemical pathway involving elevation of cAMP levels and phosphorylation of VASP. Based on these data, we propose that PDE1 inhibition may reduce inflammatory responses through reductions in cellular motility.

## 2. Material and methods

### 2.1. Cells

BV2 cells were obtained from the Banca Biologica e Cell Factory in Italy and were grown in RPMI media supplemented with 10% heat-inactivated fetal bovine serum (FBS) and 1 mM l-glutamine. Serum was decreased to 2% for 48 h before experimentation.

### 2.2. BV2 treatment conditions

Cells were pretreated for 1 h with drug treatment. ITI-214 was synthesized at Intra-Cellular Therapies, Inc. (Li et al., 2016). Other drugs were purchased from Sigma-Aldrich (ADP, IBMX, rolipram) or Tocris (AR-C 66096, and AR-C 69931). Where indicated, following pretreatment, LPS (from E. coli 0111:B4, Millipore-Sigma) was added to the media at 50 ng/mL for 4 h. At 5 h, the cells or media were harvested and processed as indicated below.

### 2.3. BV2 TNF ELISA

The concentration of TNF in the BV2 cell conditioned media following treatment was measured using the ThermoFisher mouse TNF ELISA kit, a colorimetric sandwich ELISA, following the manufacturer's instructions. Values were interpolated from a standard curve of TNF and analyzed using Excel and GraphPad Prism software.

### 2.4. RNA-Seq

BV2 cells (n = 4 cultures/treatment) were treated as described. Cells were scraped, pelleted, and flash frozen in liquid nitrogen and sent for RNA-Seq transcriptome analysis. Genewiz (https://www.genewiz.com/) performed the RNA isolation, library preparation via polyA selection, and 1 × 50bp single read sequencing on the Illumina HiSeq2500 in High Output mode (using V4 chemistry). An average of 17 million reads were obtained for each sample. Fragments were mapped to a reference mouse genome GRCm38 using CLC Genomics Server and differential gene expression analysis was performed using the software, edgeR (Bioconductor.org). DEG was determined using an ANOVA (p < 0.05) and expression level (removed genes where fewer than 2 samples have at least 1 read). Gene ontology analysis was generated using the AmiGO software version: 2.4.24 running PANTHER (PANTHER overrepresentation test release 2016-07-15) and gene ontology database released 2016-10-27. The reference list was *Mus musculus* and included all genes in the database. Biological processes were chosen using a Bonferroni correction (p < 0.05) and an enrichment score > 2.0, and only the Headers in the hierarchical view are shown.

### 2.5. RT-qPCR Array cards

Following treatment, the cells were rinsed once with PBS on ice, then harvested in Buffer RLT with 2-Mercaptoethanol according to the RNAeasy mini kit (Qiagen). Cells were scraped in the buffer, then homogenized ten times with a 21-gauge syringe. Samples were then processed according to kit instructions. RNA concentration was measured with NanoDrop spectrophotometer using samples of RNA diluted in 10 mM Tris buffer. Synthesis of cDNA was performed using the Superscript IV kit (ThermoFisher) using a starting amount of 1 μg RNA. Custom 384-well, 8-port TaqMan Low Density Arrays (TLDA microfluidic cards) were supplied by ThermoFisher. The list of genes included on the card is provided in Supplementary Table S1. One sample was loaded per port at 100 ng converted RNA each, with TaqMan Universal Master Mix II, no UNG. Cards were loaded and sealed as instructed. Each well reaction volume was 1 μL. qPCR was run on the QuantStudio 7 instrument (ThermoFisher). The following temperature run was used: 50 °C for 2 min, 95 °C for 10 min, 40 cycles of 95 °C for 15 s, 60 °C for 1 min. Ct values were calculated and normalized to the average of three reference genes, GAPDH, Ube2d2a, and Eif4a2, and vehicle control samples (ΔΔCt). Statistical analysis was performed using a one-way ANOVA with the Bonferroni post-test. All data are displayed as mean ± SEM.

### 2.6. Chemotaxis assay

We used the CytoSelect™ 96-Well Cell Migration Assay (Cell Biolabs, Inc.). The 96-well plate has a feeder tray (lower chamber) for chemoattractant (100 μM ADP) or control (serum free media), and a fitted 96 well migration tray of Boyden chambers with 5 μm pores (upper chamber) for the cells. ITI-214 was included in the upper chamber at the time of cell plating with 220,000 cells/100 μL added per well. The plate was incubated at 37 °C for 4 h. Cells attached to the underside of the upper chamber were detached and pooled with the cells in the lower chamber. The total number of cells was measured using CyQuant GR dye incubated at room temperature for 20 mins and read under the Envision fluorescence reader at 480 nm/520 nm. The relative fluorescence units (RFU) were first normalized by removing the background as measured from wells containing only solution and no cells. Values were normalized to averages of the minimum migration (Vehicle) samples, set at 0%, and maximum migration (100 μM ADP) samples, set at 100%. The dose response curve was fitted with a logarithmic, four parameter non-linear regression curve.

### 2.7. cAMP ELISA

BV2 cells were treated with ITI-214 for 30 min then stimulated for 5 min with 100 μM ADP. The media was replaced with 0.1 M HCl and the cells incubated at room temperature for 20 min before being collected by scraping and fully lysed by sonication. Samples were then centrifuged at 1000 × *g* for 10 min to remove precipitated proteins. The sample supernatant was diluted 1:2 in ELISA buffer and run in the cAMP select ELISA (Cayman Chemical). The plate was developed and read at 412 nm using a Spectramax spectrophometric plate reader running SoftMax 4.8 software (Molecular Devices, Sunnyvale, CA). Each data point was converted to % B/B0 (100 * [(sample or standard OD – average non-specific binding) / (average maximum binding – average non-specific binding)]). Standards provided were plotted and data fit to a 4-parameter logistic equation. The concentrations of the samples were interpolated from the standard curve using Microsoft Excel and GraphPad Prizm (GraphPad Software Inc., San Diego, CA). Data was normalized to the number of cells in a representative dish.

### 2.8. Phosphoprotein immunoblotting

BV2 cells (or P2 rat microglia) were treated with indicated concentrations of ITI-214 for 30 min then stimulated for 5 min with 100 μM ADP. After one wash with PBS, cells were harvested directly into 1× sample buffer (1% SDS, 4× Laemmli sample buffer) (BioRad), scraped, sonicated, and boiled for 5 min. Samples were loaded at 30 μg protein per lane on a precast 10% Bis-Tris SDS-PAGE gel (BioRad) then transferred to nitrocellulose membrane. Blots were blocked with a 1:1 solution of PBS and Odyssey buffer (LiCor) then incubated with primary antibody solutions overnight at 4 °C. The following primary antibodies, all from Cell Signaling Technologies (Danvers, MA), were used: phospho-VASP (Ser157) (#3111, 1:500), anti-VASP (9A2) (rabbit monoclonal #3132, 1:1000), anti-β-Actin (8H10D10, monoclonal #3700, 1:1000). Following washing, membranes were incubated with secondary antibodies to mouse (AlexaFluor680, Molecular Probes, Eugene, OR, Ex633Em780) or rabbit (700 nm, Rockland Immunochemicals, Gilbertsville, PA, Ex778 Em795) IgGs for 1 h at room temperature. Signals were detected and analyzed using LiCor Odyssey infrared fluorescence detector and software.

### 2.9. Microglia culture

Primary microglia isolated from P2 rat pups (methods based on (Brewer et al., 1993; Brewer and Price, 1996) and media were purchased from BrainBits, LLC (Springfield, IL), shipped overnight on cold packs in HEB: Hibernate E, B27 supplement, and GlutaMax. Cells were then warmed in 30 °C bath for 1 min, pelleted at 200 ×g for 1 min, resuspended in media (NbActiv1: Neurobasal, B27 supplement, Glutamax), and plated at a density of 16,000 cells/cm^2^ on 2 μg/mL collagen IV coated slides or plates. After 1 h, the media was replaced with enriched media containing NbActiv1 with 2 ng/mL TGFb2, 100 ng/mL IL34, 1.5 μg/mL cholesterol (based on Bohlen et al., 2017). Cells were maintained for 12 days in culture before treatment at which point ~90% of visualized cells stained positive for Iba1, indicating a microglial phenotype (see Supplementary Fig. S1).

## 3. Results

The effect of PDE1 inhibition on pro-inflammatory effects of LPS was examined using ITI-214 as detailed below.

### 3.1. PDE1 inhibition attenuates LPS-induced TNF release in BV2 cells

**Fig. 1.**
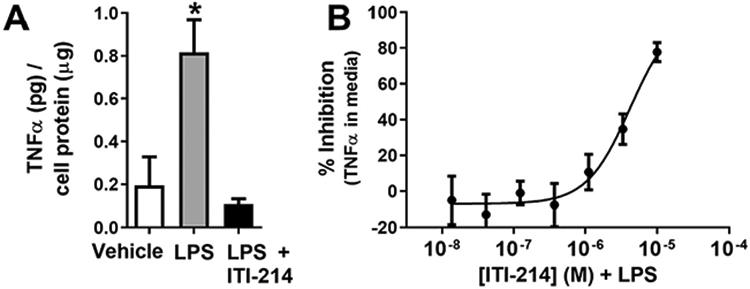
ITI-214 prevents LPS-induced release of TNF from BV2 cells. Release of TNF from treated BV2 cells was measured by ELISA and normalized to cell protein levels. (A) BV2 cells were treated with 10 μM ITI-214 then stimulated with 50 ng/mL LPS and conditioned media was analyzed (one-way ANOVA, *p < 0.05, vehicle n = 16, LPS n = 31, LPS + ITI-214 n = 14). (B) The dose dependency of the effects of ITI-214 on LPS-induced TNF release. LPS was used at 50 ng/mLl. Amount of TNF was plotted as % inhibition of total LPS response per experiment (n = 14).

BV2 cells are a cell line derived from mouse microglia with a well-characterized response to the immunogen, LPS (Blasi et al., 1990) and have been previously used to model certain microglia functions in vitro. As an initial step, we determined the ability of ITI-214 to reduce the release of the pro-inflammatory cytokine, TNF, into the media from cultured BV2 cells. TNF protein released from cultured BV2 cells was significantly increased after a 4 hour incubation with 50 ng/mL LPS. This increase was prevented by addition of 10 μM ITI-214 1 h before LPS addition (Fig. 1A). Inhibition of LPS-induced TNF release from the BV2 cells by ITI-214 was concentration dependent (Fig. 1B). This indicated that PDE1 inhibition may modulate inflammatory responses by altering release of proinflammatory cytokines.

### 3.2. Transcriptome analysis of PDE1 inhibition on BV2 cells with and without LPS stimulation

Based on this initial finding, and to more broadly explore the inflammatory pathways impacted by PDE1 inhibition, we examined the response to LPS in BV2 cells upon ITI-214 treatment using sequencing of gene transcripts (RNA-Seq) to measure changes in mRNA expression. BV2 cells (n = 4 wells/condition) were treated with vehicle, 50 ng/mL LPS, 10 μM ITI-214, or 50 ng/mL LPS plus 10 μM ITI-214, and RNA-Seq was performed as described in the Material and methods.

**Fig. 2.**
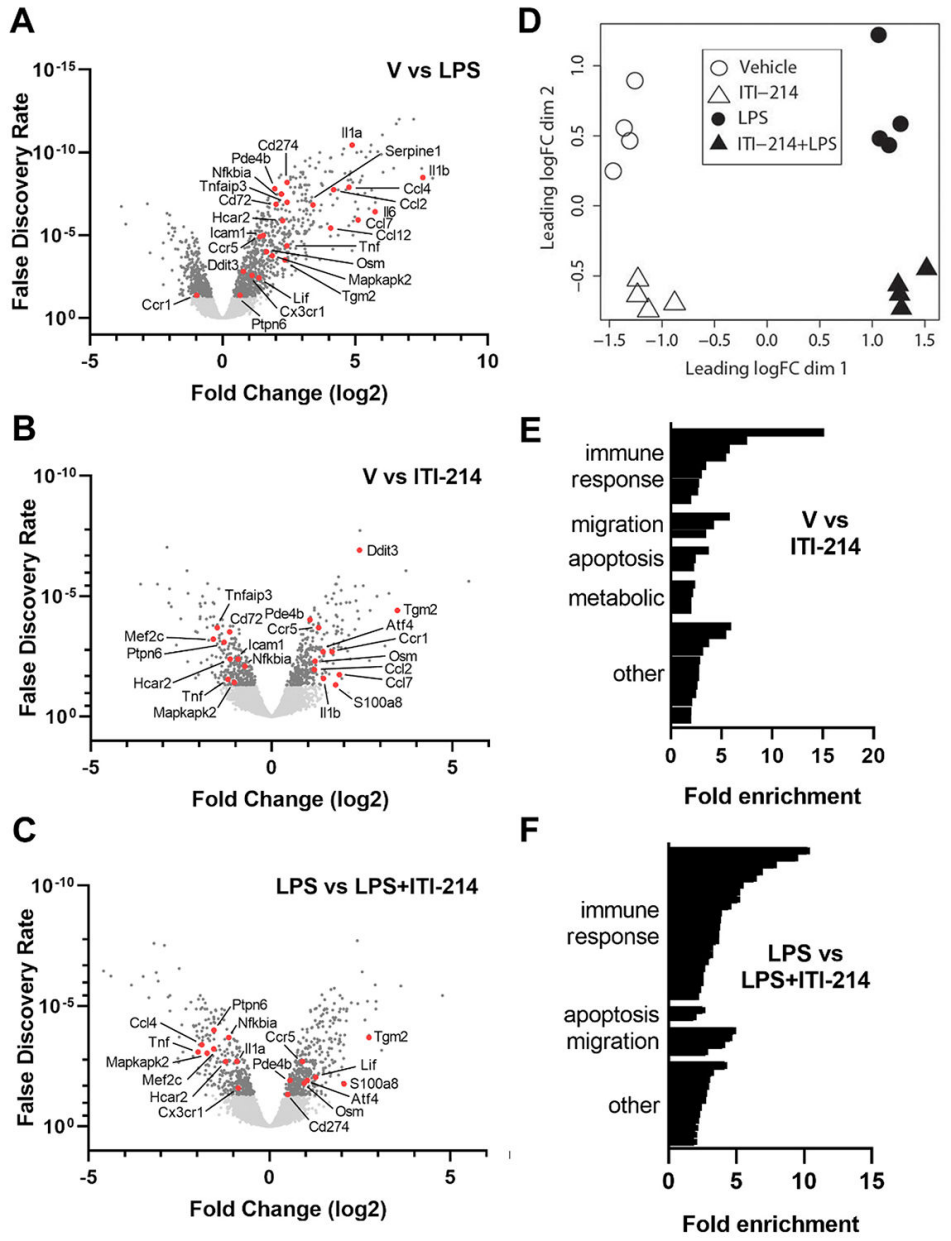
ITI-214 has transcriptome wide effects on BV2 cells at baseline and treated with LPS. The following conditions were compared in an RNA-Seq study (n = 4): Vehicle, 10 μM ITI-214, 50 ng/mL LPS, and 50 ng/mLLPS + 10 μM ITI-214. Cells were pretreated with ITI-214 for 1 h then treated with LPS for 4 h. (A–C) Volcano plots represent the differentially expressed genes from each comparison of treatments (A) Vehicle vs LPS, (B) Vehicle vs ITI-214, (C) LPS vs LPS + ITI-214. Gene changes with a false discovery rate below 0.05 are shown in dark gray. Labeled genes (shown in red) were studied in more detail with qPCR and designated the select gene panel. (D) PCA analysis of RNA-Seq study comparing BV2 cells treated with vehicle (open circles), LPS (black circles), ITI-214 (open triangles), and LPS + ITI-214 (black triangles). MDS plots were computed by edgeR software. (E–F) Graph showing the fold enrichment of each biological process from the gene ontology analysis of the treatment comparison (E) vehicle vs ITI-214 and (F) LPS vs LPS + ITI-214. The detailed breakdown of the classification of the individual processes, their fold enrichment and p-values are in Supplementary Table S3.

From the 48,440 mapped genes in the *Mus musculus* [GRCm38] database, a dataset of 11,155 expressed transcripts was identified in untreated BV2 cells, consisting of genes that yielded at least 1 RNA-Seq read in at least 2 samples. Comparison of the LPS-treated BV2 cell transcriptome to vehicle-treated cells identified 1275 differentially expressed genes (DEGs), defined as gene transcripts that showed a log2 fold change in expression >0.4 (up or down) with a false discovery rate of < 0.05 (Fig. 2A; all DEG data sets are in Supplementary Table S2 online). Gene expression changes after LPS treatment ranged from ~60-fold increases (Csf3, IL1β) to a 14-fold decrease (cytohesin 1 interacting protein, CYTIP).

As diagrammed in Fig. 2D, we observed significantly elevated expression of inflammatory genes following LPS treatment as compared with vehicle. Treatment with ITI-214 had distinct effects on both LPS-induced gene expression and basal (vehicle treatment) expression of inflammation-related genes. Statistical analysis of the DEG data sets using principal component analysis (PCA) revealed a clear separation in the pools of genes affected by each of the four treatments: vehicle, LPS alone, ITI-214 alone, and LPS + ITI-214.

ITI-214 treatment of BV2 cells yielded 710 DEGs relative to vehicle (Fig. 2B), with fold changes ranging from a ~30-fold increase (Ctla2a) to a 14-fold decrease (RNase 6). Gene ontology analysis of these DEGs indicated enrichment for genes associated with processes related to immune responses, including toll receptor signaling and cellular responses to LPS. In addition, genes involved in processes related to chemotaxis and migration were overrepresented (Fig. 2E; details in Supplementary Table S3).

**Fig. 3.**
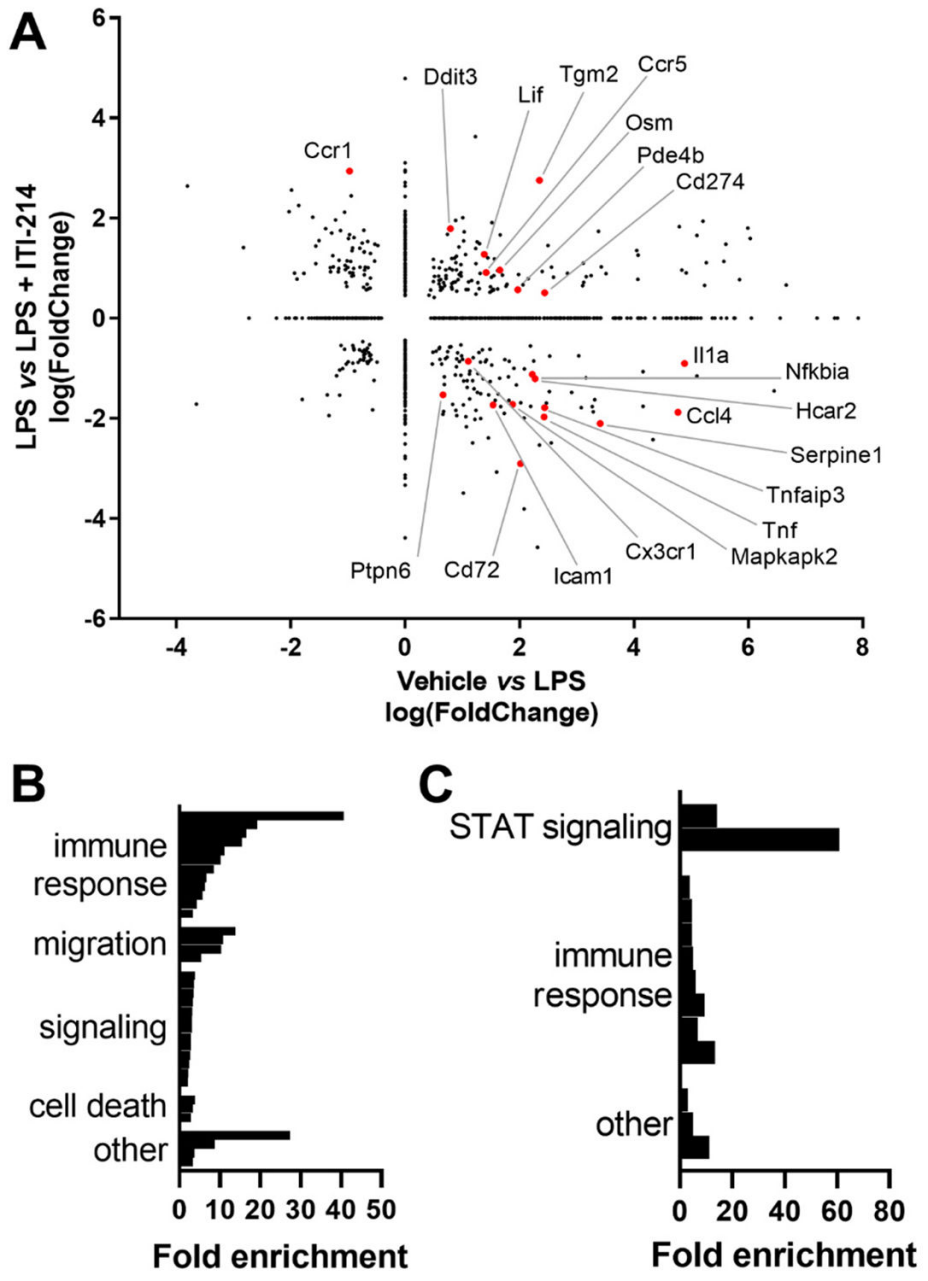
A breakdown of DEGs induced by LPS treatment and changed by pretreatment with ITI-214. (A) The fold change of the differentially expressed genes from comparing treatments vehicle vs LPS are plotted against those from LPS vs LPS + ITI-214. As in Fig. 2, the labeled genes (shown in red) were studied in more detail with qPCR. The gene ontology data from the genes that change with LPS and are (B) reversed by treatment with ITI-214 (upper left and lower right quadrants) and the genes (C) amplified by treatment with ITI-214 (lower left and upper right quadrants) are shown summarized by category. The detailed breakdown of the individual processes, their fold enrichment and p-values are in Supplementary Table S3.

The role of ITI-214 as an anti-inflammatory agent is most directly illustrated by its ability to change LPS-induced responses. Cells pretreated with ITI-214 before LPS differentially expressed 817 genes relative to those stimulated with LPS alone (Fig. 2C). The largest increase was 27-fold (Ctla2a) and the largest decrease was 24-fold (Hp, haptoglobin). The majority of the observed gene ontology processes were associated with immune responses and some with cellular migration (Fig. 2F; details in Supplementary Table S3). To put this in perspective, of the 1275 DEGs changed with LPS treatment, 332 of them were affected by addition of ITI-214 (Fig. 3A). This population of DEGs contained two main groups: (1) those genes for which LPS-induced expression change shifted towards the unstimulated state by the addition of ITI-214 (upper left and lower right quadrants) and (2) those genes for which expression change with LPS was amplified by the addition of ITI-214 (lower left and upper right quadrants). We used gene ontology analysis to identify biological processes represented by LPS-responsive transcripts reversed (Fig. 3B) or amplified (Fig. 3C) by ITI-214 pretreatment. Fewer processes were associated with the amplified group (details Supplementary Table S3).

Prior work has demonstrated inhibition of inflammatory responses to immunogens by inhibitors of cAMP-specific phosphodiesterase 4 (PDE4) (Hedde et al., 2017; Pearse and Hughes, 2016). ITI-214 is a potent inhibitor of PDE1 isoforms but also a weak inhibitor of PDE4 (Li et al., 2016). In a separate experiment, we examined the transcriptional effects of rolipram pretreatment on BV2 responses to LPS. While there are overlaps in the gene expression profiles of the two inhibitors, principal component analysis reveals that PDE1 inhibition by ITI-214 prompts a distinct gene expression pattern (Supplementary Fig. S2).

### 3.3. Dose dependent effects on select gene panel

Guided by our BV2 RNA-Seq results, we selected a gene expression panel representing a select set of 48 genes that provide a gene expression signature of ITI-214. This array was then used to further characterize the responses of each gene to a range of ITI-214 doses. Genes were selected for the array card according to the following criteria: (1) DEGs in the RNA-Seq experiment, (2) expressed in mouse brain microglia at a level > 10 FPKM as measured in Zhang et al. (2014), (3) highly expressed in microglia in comparison to other brain cells (Zhang et al., 2014), and (4) found in one or more of the processes in the GO results. Other genes that did not satisfy the above criteria were selected based on scientific literature linking them to microglial and astrocyte activation. Hereafter we will refer to the genes chosen for the array card as the Select Gene Panel.

**Fig. 4.**
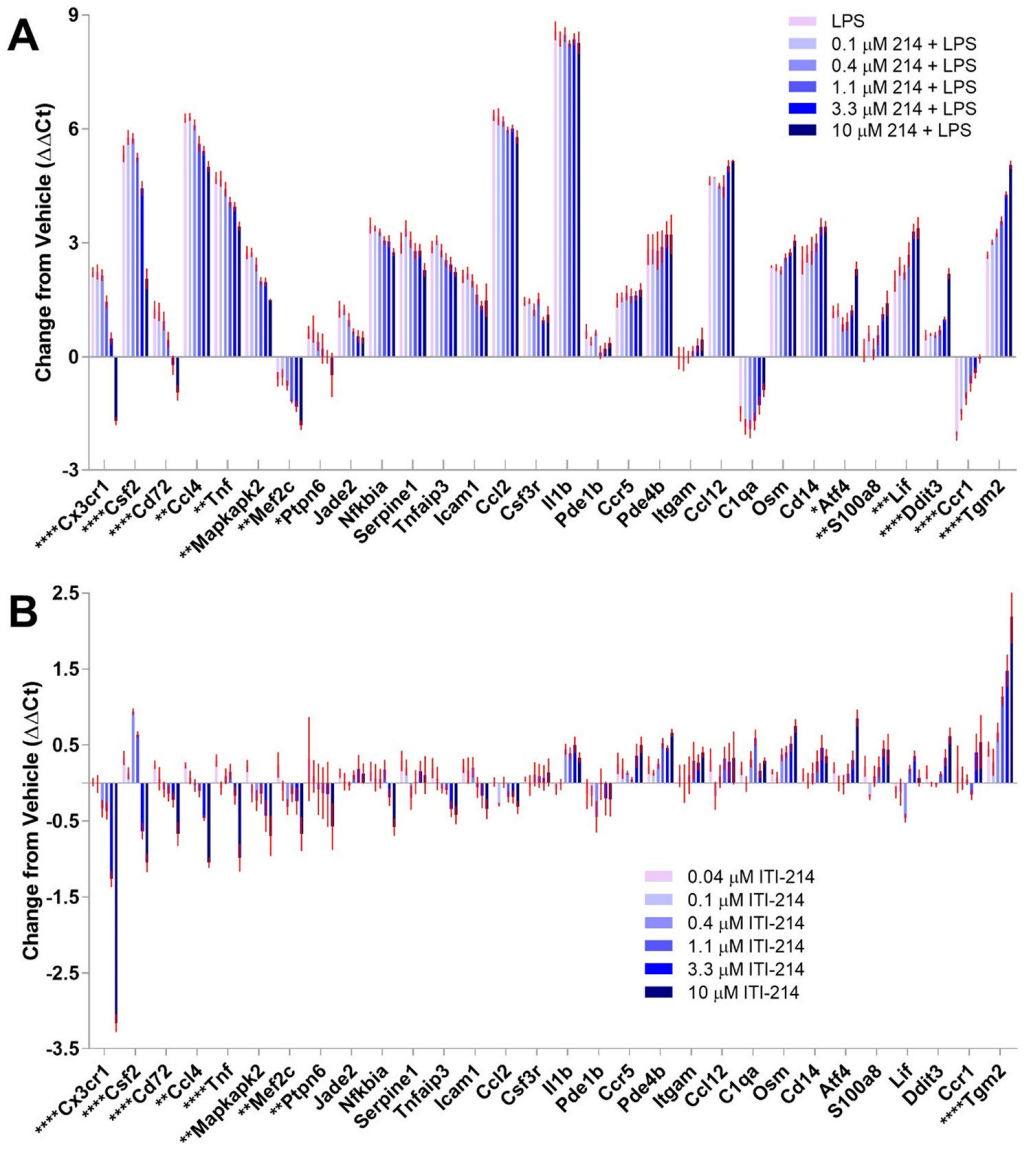
A subset of the Select Gene Panel shows dose dependent changes with ITI-214 in BV2 cells. (A) Bar graph shows the change in expression levels of each gene in BV2 cells with 50 ng/ml LPS and the indicated dose of ITI-214 (abbreviated 214). (B) The dose dependent effects of ITI-214 in the absence of LPS stimulation are shown in the bar graph. All sample values were normalized to an average of 3 reference genes and to vehicle control (ΔΔCt). The red error bars represent the mean ± SEM (n = 4). Genes listed in Supplementary Table S2 online that were expressed at very low levels in all treatments or did not show change across treatments are not shown. Significant changes between LPS and 10 μM ITI-214 + LPS (A) and Vehicle and 10 μM ITI-214 (B) are marked on the X-axis gene names and were calculated using a one-way ANOVA. * p < 0.05, **p < 0.01, ***p < 0.001, ****p < 0.0001.

We compared selected gene expression after treating BV2 cells with LPS in the presence of varying doses of ITI-214 using the Select Gene Panel. The modulation of transcriptional responses to LPS by ITI-214 that we had observed at 10 μM was dose dependent (Fig. 4A). We also treated BV2 cells with varying doses of ITI-214 without LPS. We observed that without the baseline of large LPS-driven changes in gene expression, the effect of ITI-214 was muted, but the dose dependent pattern of expression changes was largely preserved (Fig. 4B).

### 3.4. PDE1 inhibition attenuates BV2 cell migration towards a chemotactic signal

**Fig. 5.**
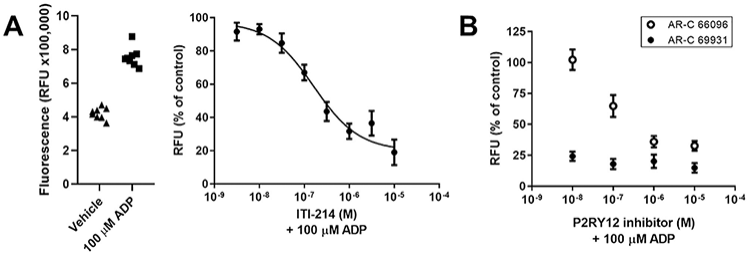
ITI-214 inhibits ADP induced migration of BV2 cells in a dose dependent manner. BV2 cells were added to upper chamber of a 5 μm pore Boyden chamber with 100 μM ADP or vehicle in the lower chamber and incubated at 37°C with 5% CO_2_ for 4 h. Indicated doses of ITI-214 were included in the upper chamber at time 0. (A) Raw fluorescence counts (relative fluorescence units or RFU), representing the cell count in the lower chamber, for the control conditions. The Vehicle condition is the minimum migration; there is no ADP present. The maximum migration is the 100 μM ADP condition. The average of each control condition in the left graph was used to normalize the data from the ITI-214 treated samples. The curve was fit to a 4-parameter logarithmic equation with the following constraints: top > 80% and bottom < 20%. The resulting IC50 is 0.16 μM. n = 8 (B) Dose dependent inhibition of ADP chemotaxis by P2Y12 inhibitors. AR-C 66096 (*open circles*; documented IC50 of 6.9 nM) and AR-C 69931 (*black circles*, documented IC50 of 0.4 nM) inhibit ADP induced migration in a Boyden chamber system.

Since RNA-seq analysis of ITI-214 effects on BV2 cells (and BV2 responses to LPS) revealed gene ontology processes associated with chemotaxis and migration, we measured cell migration directly using ADP as a chemotactic signal. ADP is a known chemoattractant for microglia, released from and acting as a signal for the presence of dead or dying neurons (Honda et al., 2001), and BV2 cells will migrate towards this signal. Using a Boyden chamber, we plated cells in the upper chamber and measured the migration of cells into the lower chamber containing 100 μM ADP (Fig. 5A, left panel). ITI-214 reduced chemotaxis of BV2 cells towards 100 μM ADP in a dose-dependent manner (Fig. 5A, right panel). Blockade of the P2Y12 purinergic receptor, by AR-C 66096 (IC_50_ is 6.9 nM; Humphries et al., 1994) or AR-C 69931 (IC_50_ is 0.4 nM; Ingall et al., 1999) also attenuated ADP chemotaxis in BV2 cells, indicating an important role for this receptor in the migration response (Fig. 5B).

### 3.5. PDE1 inhibition augments ADP-dependent cAMP and phospho-VASP (Ser157) responses

**Fig. 6.**
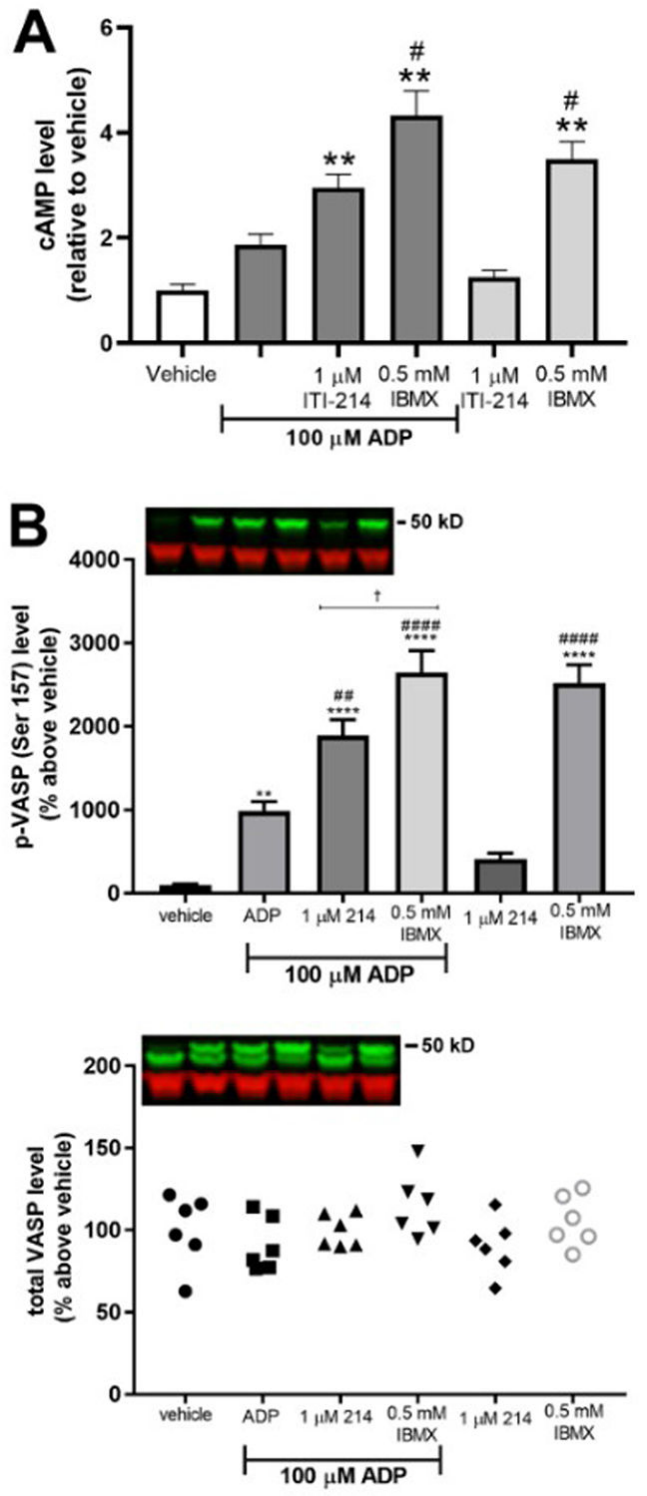
ITI-214 enhances ADP-induced cAMP and p-VASP (S157) levels in BV2 cells. BV2 cells were treated with ITI-214 or IBMX for 30 min prior to stimulation with ADP for 5 min. (A) Cyclic AMP levels were normalized to the vehicle value of pmol cAMP/number of cells. (B) Levels of phosphorylation of VASP at serine 157 were measured on a Western blot. The top graph shows phosphorylation and the bottom graph shows levels of total VASP. Insets show representative samples; the same samples were loaded on each blot in the order indicated in the bar graphs. Green bands are p-VASP (top) or total VASP (bottom). Samples were normalized to actin as a loading control (red bands). Data was normalized to vehicle and statistical significance was determined with an ANOVA. *Relative significance*: *p < 0.05, **p < 0.01, ***p < 0.001, ****p < 0.0001 *compared to vehicle alone;* ^#^p < 0.05, ^##^p < 0.01, ^###^p < 0.001, ^####^p < 0.001 *compared to ADP alone;* ^†^p < 0.05 *between ITI-214* + *ADP and IBMX* + *ADP*.

The ADP chemotactic response in BV2 cells involves a signaling cascade driven by P2Y12 receptors, as shown graphically in Supplementary Fig. 3 (Fig. S3). Receptor binding triggers increases in cAMP through stimulation of adenylyl cyclase via a cell-specific receptor-coupled stimulatory G-protein. This leads to increased PKA activity, which phosphorylates VASP. As shown (and stated) by Lee and Chung (2009), “VASP phosphorylation by PKA plays an important role in membrane ruffle formation and chemotaxis via the regulation of focal adhesion formation/maturation.” While ADP has been shown to stimulate cell migration via increased cAMP and the subsequent phosphorylation of VASP (serine 157) in BV2 cells, prolonged high levels of cAMP and VASP phosphorylation (via forskolin treatment or phosphatase inhibition) inhibit migration (Lee and Chung, 2009). Inhibition of cAMP breakdown might be expected to have the same effect on cAMP, phospho-VASP, and therefore ADP-dependent chemotaxis as forskolin treatment. To test this hypothesis we pretreated BV2 cells with ITI-214 (1 μM for 30 min) prior to addition of ADP (100 μM, 5 min). ITI-214 pretreatment significantly augmented cAMP levels compared to ADP alone or vehicle (n = 6, Fig. 6A). ITI-214 treatment in the absence of ADP stimulation had no effect on cAMP levels [compare with the pan-PDE inhibitor, 3-isobutyl-1-methylxanthine (IBMX), which increased cAMP levels even at baseline (Fig. 6A)] Cyclic AMP-dependent phosphorylation of VASP at serine 157 showed an even more striking increase under the same treatment conditions (n = 6, Fig. 6B). Levels of p-VASP in the presence of ADP were significantly higher than with vehicle and the level increased significantly above ADP alone upon ITI-214 pretreatment. As with cAMP, PDE1 activity (sensitive to ITI-214) appears to contribute about 50% to the total PDE activity (sensitive to IBMX) under conditions of stimulation while having no contribution at baseline.

### 3.6. Phospho-VASP (Ser157) responses of primary microglial cells

**Fig. 7.**
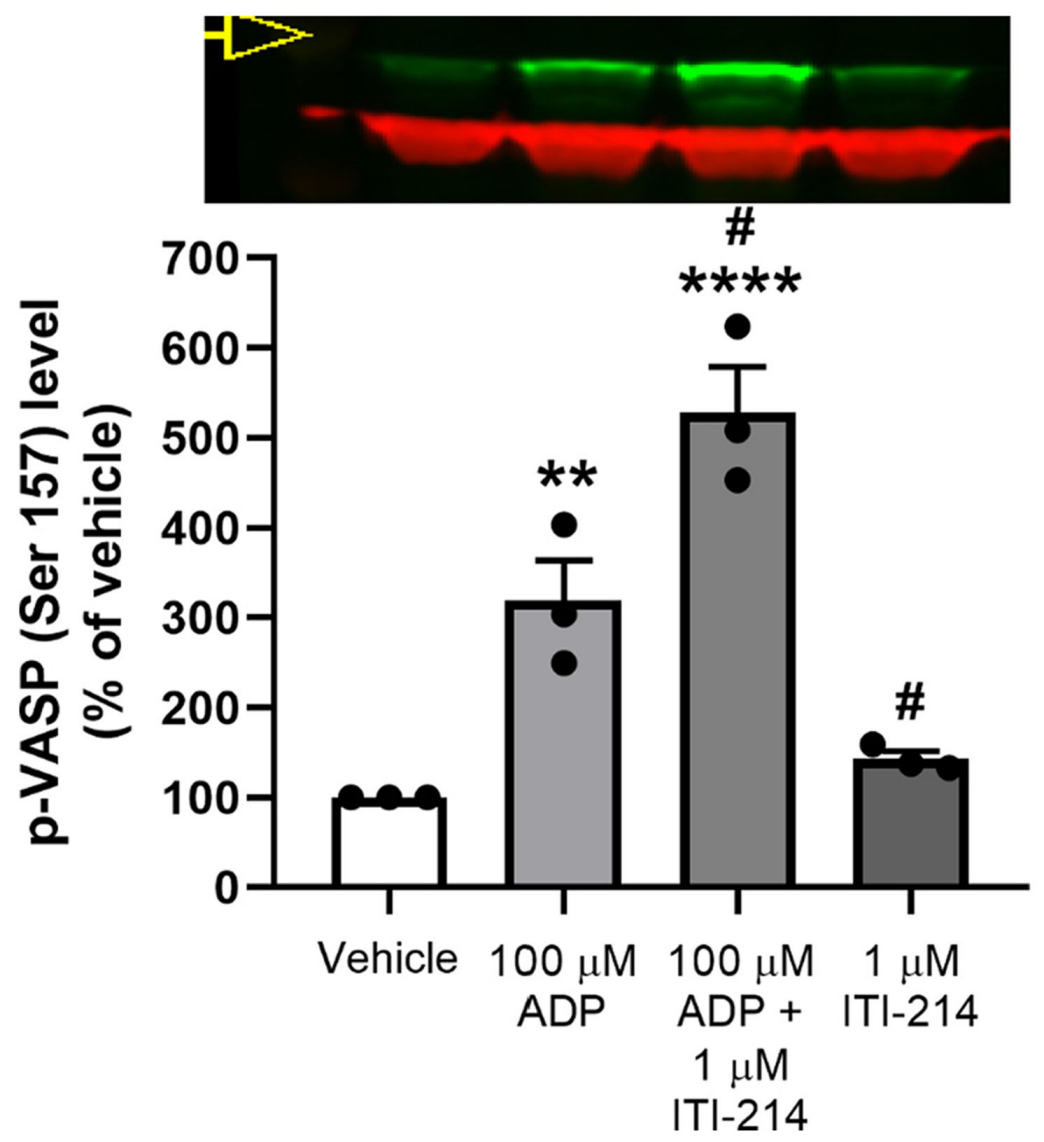
ITI-214 enhances ADP-induced p-VASP (S157) levels in cultured primary rat microglia. Microglia were treated with ITI-214 for 30 min prior to stimulation with ADP for 5 min. (A) Levels of phosphorylation of VASP at serine 157 were measured on a Western blot. Insets show representative samples; the same samples were loaded on each blot in the order indicated in the bar graphs. Green bands are p-VASP (top) or total VASP (bottom). Samples were normalized to actin as a loading control (red bands). Data was normalized to vehicle and statistical significance was determined with an ANOVA. *Relative significance:* **p < 0.01, ****p < 0.0001 *compared to vehicle alone,* ^#^p < 0.05 *compared to ADP alone*.

The above results were replicated in primary cultured microglia isolated from P2 rat whole brain (Fig. 7). Microglia were grown in culture for 12 d in serum-free media containing TGFb2, IL34, and cholesterol (Bohlen et al., 2017). These cells maintained properties in culture consistent with quiescent microglial-like cells, staining positive for Iba1 and maintaining a branched morphology (see Material and methods and Supplementary Fig. 1). As with BV2 cells, addition of 1 μM ITI-214 significantly enhanced the increase in VASP phosphorylation detected after treatment with 100 μM ADP for 5 mins, while having little effect under basal conditions (n = 3) (Fig. 7). Replication of these data in microglia cells suggests that findings in the BV2 cells may be representative of microglial cellular responses.

## 4. Discussion

ITI-214 is a potent and specific inhibitor of calcium/calmodulin dependent phosphodiesterases (the PDE1 family) and the first to be tested in humans, where it has proven to be safe and well tolerated in normal healthy volunteers and patients with Parkinson's disease. It has documented roles in central nervous system (Snyder et al., 2016) and cardiovascular function (Hashimoto et al., 2018), where inhibition of PDE1 may control responses under conditions of elevated intracellular calcium. ITI-214 exhibited a dose-dependent anti-inflammatory action in LPS-treated BV2 cells, downregulating certain inflammatory cytokines and gene expression related to chemotaxis. Functional assays confirmed that ITI-214 dose dependently inhibited migration in response to ADP via amplified signaling through cAMP and, subsequently, increased phosphorylation of VASP.

As an anti-inflammatory agent, ITI-214 significantly attenuated release of the key inflammatory mediator, TNF. TNF is upregulated in multiple models of neurotoxicity and disease and is reversed with known anti-inflammatory agents (Little et al., 2002). Increased TNF is associated with Alzheimer's and Parkinson's disease and disruption of TNF and its receptor is protective to dopaminergic neurons in Parkinson's disease (McCoy et al., 2006). ITI-214 also attenuated LPS-induced expression of Ccl2 mRNA. Decreased levels of Ccl2 have been shown to be protective in models of traumatic brain injury and intracerebral hemorrhage (Gyoneva and Ranshohoff, 2015; Yang et al., 2016).

Importantly, the pattern of gene expression changes observed with ITI-214 was distinct from changes observed upon cell treatment with the PDE4 inhibitor, rolipram. Based on the observed activity of ITI-214 versus recombinant PDE4A (IC_50_ = 0.160 μM) (Li et al., 2016), PDE4 inhibition could contribute, but cannot be solely responsible for the effects of ITI-214 in the cells studies here.

Transcriptome analysis with RNA-Seq revealed a pattern of gene expression changes in BV2 cells in response to ITI-214 under basal and LPS-stimulated conditions consistent with an overall anti-inflammatory effect. Within the ITI-214 transcriptome we identified other genes in BV2 cells that responded in concert with inflammatory markers, including genes encoding intracellular enzymes that control microglial reactivity, such as Mapkapk2 and PtpN6. Expression of both Mapkapk2 and PtpN6 genes was significantly elevated by LPS and diminished by ITI-214 treatment. Mapkapk2 has been implicated as an effector in inflammatory responses leading to neuronal damage and cell death in various disease models. For instance, gene knockout of Mapkapk2 improves functional recovery of animals after spinal cord injury (Ghasemlou et al., 2010), prevents neuronal death induced by amyloid beta 1–42 in a model of Alzheimer's disease (Culbert et al., 2006), and prevents neuronal death in the MPTP model of Parkinson's disease (Thomas et al., 2008). Our data would indicate that inhibition of PDE1 activity, in addition to dampening inflammatory responses, may also mediate neuroprotective actions by suppressing activity of enzymes like Mapkapk2 and PgpN6 that drive cellular pathways resulting in neuronal injury and death.

Based on these RNAseq results, we created a qPCR gene expression panel to monitor the response of several microglial-expressed genes under conditions of elevated cyclic nucleotide signaling and at various doses of ITI-214. Of the genes we chose for expanded analysis, *Cx3cr1* and *Tgm2* showed the most dramatic change upon ITI-214 treatment. Induction of Cx3cr1 by LPS was significantly reduced by ITI-214 treatment, as was basal expression of this marker in the absence of LPS. Cx3cr1, a receptor for the chemotactic cytokine, Cx3cl1 (or fractalkine), is elevated by inflammatory events occurring as a result of a variety of neuronal insults. For example, Cx3cl1 is elevated by LPS, HIV infection, as well as in brain tumors, epilepsy, and in other neuropathological conditions (Limatola and Ransohoff, 2014). Further, genetic knockout of Cx3cr1 improves recovery following spinal cord injury (Freria et al., 2017) and confers resilience to stress (Hellwig et al., 2016; Rimmerman et al., 2017; Wohleb et al., 2017). Thus, the ability of PDE1 inhibition to dampen Cx3Cl1 signaling through reduced expression of the receptor would be expected to be neuroprotective in a number of pathological states.

Tgm2 expression in the immune system has alternately been reported as either beneficial or detrimental. It is considered to be a (beneficial) M2 state monocyte marker (Morganti et al., 2016) and is upregulated by the anti-inflammatory cytokine TGFb (Fuchshofer et al., 2005; Quan et al., 2005; Tovar-Vidales et al., 2011). Tgm2 functions to crosslink extracellular matrix proteins with beneficial results in wound healing (Nanda et al., 2001; Quan et al., 2005). Detrimental influence of Tgm2 has been suggested based on the presence of N epsilon-(gamma-*glutamyl*)-lysine) cross-links in neurofibrillary tangles in Alzheimer's cases (Singer et al., 2002).

Gene ontology analysis of genes differentially expressed upon drug treatment revealed (besides expected inflammatory signals) significant enrichment in genes involved in chemotaxis, providing a novel hypothesis to test: ITI-214 should affect BV2 (and microglial) cell migration. Damaged or dying neurons lose membrane integrity and release ATP into the extracellular space which is quickly hydrolyzed to ADP, and ADP in turn acts as a chemotaxis signal, directing migration of microglia to the site of injury to phagocytize and clear cellular debris. The target receptor for ADP chemotaxis on microglia and on BV2 cells is the purinergic receptor, P2Y12 (Honda et al., 2001). This receptor is thought to be coupled to Gi/o protein, which upon receptor activation inhibits adenylyl cyclase and, thus, the formation of cAMP. In microglia, however, increased cAMP, typically associated with Gs protein, has been documented to arise from activation of P2Y12 by ADP. Further, when cAMP levels are enhanced beyond the level triggered by ADP, for example by addition of forskolin, chemotaxis towards ADP is inhibited (Fan et al., 2017). Under these conditions, activation of cAMP-dependent protein kinase (PKA) leads to phosphorylation of the focal adhesion associated protein, vasodilator stimulated phosphoprotein (VASP). VASP is an established target of PKA that has been associated with motility via its effect on actin capping and (substrategrabbing) focal adhesions. It is evident that a balance of cAMP and PKA activity, with VASP dephosphorylation controls cellular migration; in general, small increases in cAMP promote migration and larger increases interrupt migration (Howe, 2004). High levels of cAMP pathways activity may impair migration, in part, through the ability of phosphorylated VASP to promote actin capping and maintain focal adhesions (Worth et al., 2010), thus, reducing the turnover of focal adhesions and the speed of migration.

Limiting PDE1 activity via ITI-214 treatment allows a prolonged increase in cAMP levels under conditions of stimulation with ADP, and increased VASP phosphorylation in both BV2 cells and isolated microglia. We suggest that ITI-214 inhibits migration through deregulation of cAMP pathways in the microglia (Figs. 6, 7 and Fig. S3). We cannot rule out ITI-214 influences on other signaling pathways, notably cGMP, the other substrate for PDE1 enzymes: however, the cGMP levels we measured in BV2 cells and P2 rat microglia were vanishingly small under all the conditions studied. Nor can we eliminate a concurrent inhibition of PDE4 isoforms by ITI-214 as a contributory factor.

Inhibition of migration may be an effective treatment for multiple disease indications including neurodegeneration. Initially, recruitment of microglia is beneficial to the inflammatory response of a dying cell. Prolongation of the response is likely to be detrimental to neighboring neurons and synapses (Hong and Stevens, 2016). ITI-214 inhibits ADP-dependent migration of BV2 cells without affecting migration or underlying cAMP dependent signaling in the absence of the ADP trigger, and thus may serve to slow the unchecked immune response. The ability of PDE1 inhibitors to prevent or dampen excessive inflammatory responses of microglia provides a basis for exploring their therapeutic utility in the treatment of neurodegenerative diseases such as Parkinson's disease and Alzheimer's disease. Based partly on these findings, ITI-214 is currently being studied in patients with Parkinson's disease.

Supplementary data to this article can be found online at https://doi.org/10.1016/j.mcn.2019.103449.

## Supplementary Material

supp table S1

supp table S2

supp table S3

Supplemental Figures 1-3

## Abbreviations:

ADP: adenosine diphosphate
cAMP: cyclic adenosine 5′-monophosphate
cGMP: cyclic guanosine 5′-monophosphate
DEG: differentially expressed gene
GM-CSF: granulocyte-macrophage colony-stimulating factor
IL-1β: interleukin-1, beta
IL-6: interleukin-6
LPS: lipopolysaccharide
TNF: tumor necrosis factor
P2Y12: P2Y purinoceptor, 12
PDE1: phosphodiesterase-1
PDE4: phosphodiesterase-4
PKA: cyclic AMP-dependent protein kinase
RT-qPCR: real time quantitative polymerase chain reaction
VASP: vasodilator-stimulated phosphoprotein
