# Supplementary figures and images for: Inhibition of calcium-calmodulin-dependent phosphodiesterase (PDE1) suppresses inflammatory responses
Source: Mol Cell Neurosci. Author manuscript; Available in PMC 2021 Jan 5. (PMC7783477; doi:10.1016/j.mcn.2019.103449)

**Supplementary Figure 1**

**
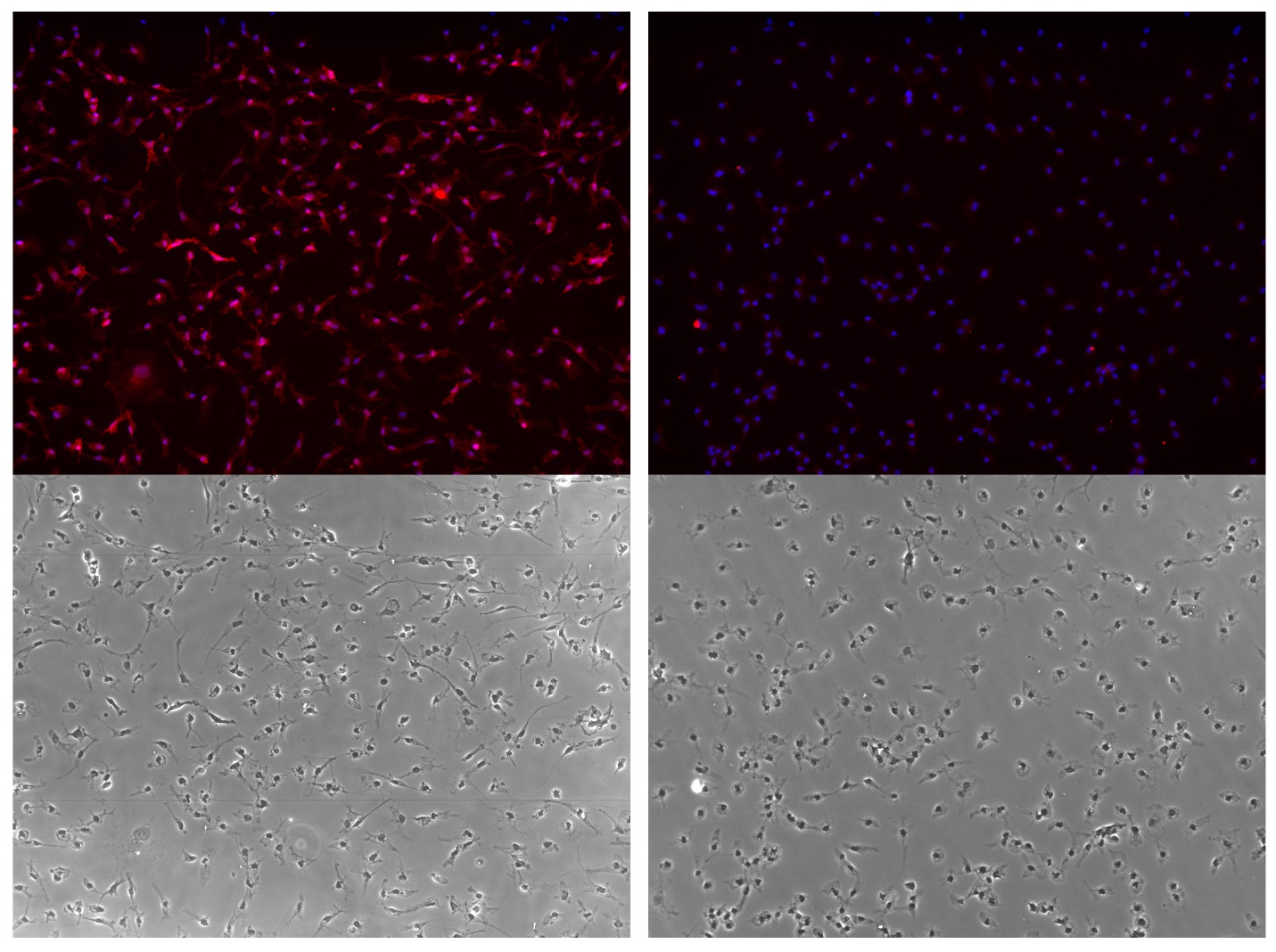
**

**Supplementary Figure S2**
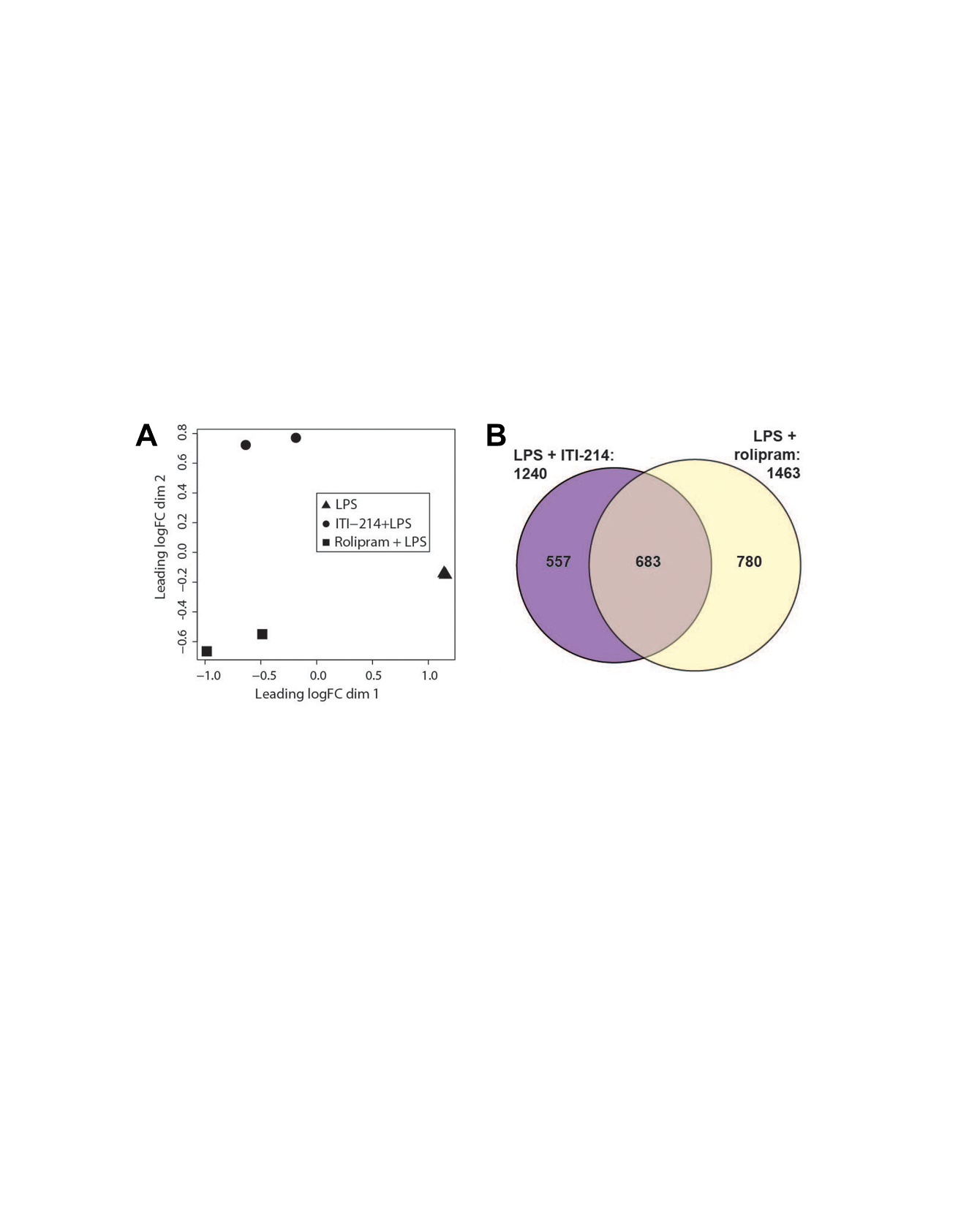


**Supplementary Figure S3**

**
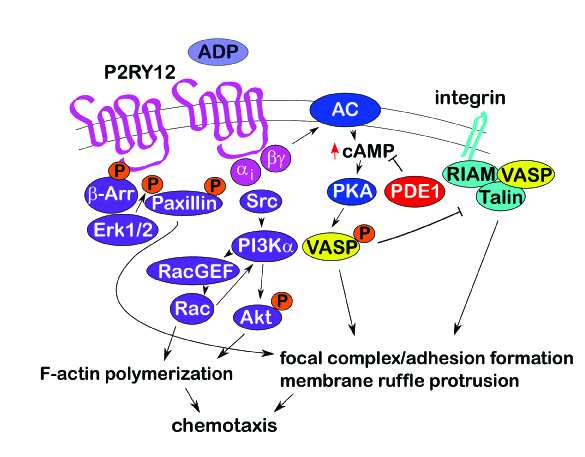
**

Supplement: Supplemental Figures 1-3 [file NIHMS1653670-supplement-Supplemental_Figures_1-3.docx]
